# Analgesia with 5' extracellular nucleotidase-mediated electroacupuncture for neuropathic pain

**DOI:** 10.1590/0004-282X-ANP-2021-0149

**Published:** 2022-03-30

**Authors:** Qin-xue Dai, Shan Li, Miao Ren, Xinlu Wu, Xin-yu Yao, Fei-hong Lin, Xu-qing Ni, Yun-chang Mo, Jun-lu Wang

**Affiliations:** 1 Wen Zhou Medical University, The First Affiliated Hospital, Department of Anesthesiology, Wenzhou, China. Wen Zhou Medical University The First Affiliated Hospital Department of Anesthesiology Wenzhou China

**Keywords:** Electroacupuncture, Analgesia, 5'-nucleotidase, Neuralgia, Eletroacupuntura, Analgesia, 5’-Nucleotidase, Neuralgia

## Abstract

**Background:**

Acupuncture is a treatment for neuropathic pain, but its mechanism remains unclear. Previous studies showed that analgesia was induced in rats with neuropathic pain when their spinal cord adenosine content increased after electroacupuncture (EA); however, the mechanism behind this electroacupuncture-induced increase has not been clarified.

**Objective:**

This study aimed to determine the role that ecto-5’-nucleotidase plays in EA-induced analgesia for neuropathic pain.

**Methods:**

We performed electroacupuncture at the Zusanli acupoint on the seventh day after establishing a rat model of neuropathic pain induced through chronic constriction injuries. We observed the mechanical withdrawal threshold and thermal pain threshold and detected the expression of ecto-5’-nucleotidase in the spinal cord using Western blot. Chronic constriction injury rat models were intraperitoneally injected with α,β-methyleneadenosine 5'-diphosphate, an ecto-5’-nucleotidase inhibitor, 30 min before electroacupuncture. The adenosine content of the spinal cord was detected using high-performance liquid chromatography. Lastly, the adenosine A1 receptor agonist N6-cyclopentyladenosine was intrathecally injected into the lumbar swelling of the rats, and the mechanical withdrawal and thermal pain thresholds were reevaluated.

**Results:**

Analgesia and increased ecto-5’-nucleotidase expression and adenosine content in the spinal cord were observed 1 h after electroacupuncture. α,β-methyleneadenosine 5'-diphosphate was able to inhibit upregulation of adenosine content and electroacupuncture-induced analgesia. After administration of N6-cyclopentyladenosine, electroacupuncture-induced analgesia was restored.

**Conclusions:**

Our results suggest that electroacupuncture at Zusanli can produce analgesia in chronic constriction injury rat models, possibly via the increased ecto-5’-nucleotidase expression induced through electroacupuncture, thus leading to increased adenosine expression in the spinal cord.

## RESUMO

**Antecedentes:**

A acupuntura é um tratamento para a dor neuropática, mas seu mecanismo permanece obscuro. Estudos anteriores mostraram que a analgesia foi induzida em ratos com dor neuropática quando o conteúdo de adenosina da medula espinhal aumentou após a eletroacupuntura (EA); no entanto, o mecanismo por trás desse aumento induzido por eletroacupuntura não foi esclarecido.

**Objetivo:**

Este estudo teve como objetivo determinar o papel que a ecto-5'-nucleotidase desempenha na analgesia induzida por EA para dor neuropática.

**Métodos:**

Realizamos eletroacupuntura no ponto de acupuntura de Zusanli no sétimo dia após estabelecer um modelo de rato de dor neuropática induzida por lesão de constrição crônica. Observamos o limiar de retirada mecânica e o limiar de dor térmica, e detectamos a expressão de ecto-5'-nucleotidase na medula espinhal usando Western blot. Modelos de ratos com lesão de constrição crônica foram injetados intraperitonealmente com α, β-metilenadenosina 5'-difosfato, um inibidor de ecto-5'-nucleotidase, 30 min antes da eletroacupuntura. O conteúdo de adenosina da medula espinhal foi detectado por meio de cromatografia líquida de alta eficiência. Por último, o agonista do receptor de adenosina A1 N6-ciclopentiladenosina foi injetado por via intratecal no edema lombar dos ratos, e a retirada mecânica e os limiares de dor térmica foram reavaliados.

**Resultados:**

Analgesia e aumento da expressão de ecto-5'-nucleotidase e conteúdo de adenosina na medula espinhal foram observados 1 h após a eletroacupuntura. α, β-metilenadenosina 5'-difosfato foi capaz de inibir a sobrerregulação do conteúdo de adenosina e analgesia induzida por eletroacupuntura. Após administração de N6-ciclopentiladenosina, a analgesia induzida por eletroacupuntura foi restaurada.

**Conclusões:**

Nossos resultados sugerem que a eletroacupuntura em Zusanli pode produzir analgesia em modelos de ratos com lesão de constrição crônica, possivelmente por meio do aumento da expressão de ecto-5'-nucleotidase induzida por eletroacupuntura, levando ao aumento da expressão de adenosina na medula espinhal.

## INTRODUCTION

Neuropathic pain has a high incidence rate and its treatment is difficult. This seriously affects the work and lives of patients and poses challenges to clinicians^1^. Therefore, the search for an effective, nontoxic treatment with minimal side effects has received close attention from the scientific community. In the late 1990s, the U.S. National Institutes of Health claimed that acupuncture is an effective alternative treatment for lumbago and leg pain^2^, but the mechanism of acupuncture analgesia remains unclear. Our previous studies showed that the adenosine content in the spinal cord of rats with chronic constriction injuries (CCIs) can significantly rise 60 min after electroacupuncture (EA), thus producing analgesic effects in these rats^2^. However, we could not determine how EA caused the adenosine content in the spinal cord of CCI rats to increase.

Previous studies have shown that adenosine triphosphate (ATP), adenosine diphosphate (ADP), adenosine monophosphate (AMP) and adenosine are all categorized as purine substances^3^^,^^4^. ATP is stripped of a phosphate group by a dephosphatase to become ADP, and ADP is then stripped of another phosphate group by a dephosphatase to form AMP. Loss of a phosphate group from AMP under the action of ecto-5’-nucleotidase (5'-NT) results in formation of adenosine. Via the action of adenosine deaminase, adenosine is then deaminated to become creatinine, which is eventually metabolized by the kidneys and excreted in urine^4^. Recent studies have suggested that 5'-NT is the key enzyme for adenosine production^5^, and it is worth exploring whether 5’-NT is the target of EA when promoting adenosine production. 

Therefore, this study investigated whether EA produces analgesic effects on CCI rats via promotion of increased spinal adenosine, through upregulating 5'-NT expression.

## METHODS

### Animals

The study protocol was approved by the Institutional Animal Experimental Ethics Committee at the First Affiliated Hospital of Wenzhou Medical University, China (approval no. 12045). The experimental animal center of Wenzhou Medical University provided 42 healthy adult male Sprague-Dawley rats (specific-pathogen-free grade) weighing 220 to 250 g for this study (animal No: 2007000517448). The rats were purchased three days before surgery and were made to fast for one day but were allowed to drink freely before surgery to prevent reflux and aspiration. 

### CCI model

In accordance with the method described by Bennett^6^, the rats were anesthetized with intraperitoneal chloral hydrate (10%; 350 mg/kg) and routinely disinfected. The femoral bicep muscles in the right lower extremities were separated, and the sciatic nerve trunks were exposed and tied at four sites at 1-mm intervals using 4-0 chromium-containing catgut. The tying force was sufficient to induce a slight leg muscle or toe jerk. The incisions were then sutured, and the rats were returned to their cages. In the sham group, the sciatic nerve was exposed, but no knots were tied, while the other procedures remained identical. After surgery, the right lower limb showed lameness, and wandering and was held in a defensive position when the rats had fully awakened from anesthesia; they would also occasionally lick the limb. These cases confirmed successful induction of chronic sciatic nerve compression. Seven rats with autophagy or wound infections were excluded and replaced.

### Experimental grouping

The 42 rats were randomly divided into seven groups with six rats in each: sham group; model group; EA group; EA + α,β-methyleneadenosine 5'-diphosphate (AOPCP) group; EA + normal saline (NS) group; EA + AOPCP + adenosine A1 receptor agonist N6-cyclopentyladenosine (CCPA) group; and EA + AOPCP + dimethylsulfoxide (DMSO) group. 

### Administration method

In accordance with the method proposed by Bennett^6^, a sterile PE-10 catheter was inserted slowly into the subarachnoid space to prevent lumbar enlargement (L_4-6_) under anesthesia, three days before establishment of the CCI model. Subsequently, lidocaine (2%; 10 μL) was injected. If the rats developed temporary paralysis of both lower extremities, the catheterization was considered successful. The rats were then observed for signs of neurobehavioral defects, such as paralysis or limping. Two rats were abandoned because they showed these symptoms, and the rejected rats were replaced.

AOPCP powder was dissolved in 0.9% sodium chloride solution, and CCPA powder was dissolved in 10% DMSO. The rats in the EA + AOPCP and EA + NS groups were intraperitoneally injected with AOPCP (20 mg/kg)^7^ and an equal amount of 0.9% sodium chloride solution, respectively, 30 min before EA. Following intraperitoneal AOPCP injection, the rats in the EA + AOPCP + CCPA and EA + AOPCP + DMSO groups were intrathecally injected with CCPA (1 mmol/L; 10 μL) and DMSO through a pre-prepared PE catheter, respectively.

### EA treatment

The Zusanli acupoint is located approximately 5 mm below the fibula head in the knee joint^8^. An acupuncture needle was used to puncture the Zusanli acupoints in the legs, followed by electrical stimulation at a current intensity, frequency and duration of 1 mA, 2/100 Hz, and 30 min, respectively.

### Mechanical pain threshold measurement

To determine the baseline value of the mechanical withdrawal threshold before experimental treatment, the rats were placed in a special transparent glass box with a grid bottom. After 30 min of silence, the rats were stimulated with filaments of different weights (0.008, 0.02, 0.04, 0.07, 0.16, 0.4, 0.6, 1, 1.4, 2 and 4) in ascending order, and their foot shrinking reaction was observed. The right hind paws were stimulated 10 times with each filament for a duration of 1 to 2 s each time, and the interval between each instance was 1 min. The number of instances of foot shrinkage was recorded, and the corresponding foot shrinkage rate (foot shrinkage rate = (foot shrinkage instance number / 10 times) × 100%) with different gram weights of filaments for all of the rats was estimated, in order to find the filaments that induced the foot shrinkage rate closest to 50%^8^. In this experiment, the shrinkage rate induced by a 0.4 g fiber was 45%. Thereafter, a 0.16 g fiber was used to measure the number of instances of foot shrinkage among the rats in each group and to calculate the foot shrinkage rate of the rats, as follows: mechanical withdrawal threshold (MWT) = 100% -foot shrinkage rate.

### Thermal pain threshold measurement

The rats were placed in a glass box with a glass plate of thickness 3 mm at the bottom. After the rats had been allowed to settle in the glass box for 20 min, the skin on the bottom of the right feet of the rats was irradiated with a claw thermal sense tester, with a cutoff time of 20 s, to prevent scalding of the rats’ feet, which is often caused by a superfluously long test duration. When the right hind limb was raised or retracted to cut off contact with the tester, the heat source was automatically disconnected, and the exposure time was recorded as the thermal pain threshold (TPT; s). The measurement was repeated three times for each rat and was repeated every 5 min, and the average value was used as the TPT of the rat^9^.

### Detection of adenosine levels through high-performance liquid chromatography

The rats’ brains were removed and stored at -80 ℃. After adding perchloric acid (0.4 mol/L; 10 mL/g) by weight, the sample was homogenized at a high speed (-4 ℃) and centrifuged (4000 r/min, 15 min). The pH value of the sample was adjusted to 6.0-7.0 with potassium hydroxide (4 mol/L), and the liquid was centrifuged again (4000 r/min; 15 min). The rest of the high-performance liquid chromatography process was the same as published in previous papers^10^.

### Detection of 5’-NT expression through western blot analysis

After the rats had been euthanized, the L_4-6_ spinal cord segments were removed, and the total protein content of the spinal cord tissue was extracted using a cellular protein extraction kit. Equal amounts of samples were transferred to a nitrocellulose (NC) membrane by means of polyvinylidene fluoride membrane protein isolates. The NC membrane was incubated with bovine serum albumin for 90 min, and then sealed with bovine serum. Rabbit anti-rat 5'-NT protein monoclonal antibodies were added, and the NC membrane was then incubated at 4 °C overnight. The secondary antibody was rinsed by shaking and incubating at room temperature, followed by chemiluminescence, development and imaging. β-actin was used as an internal reference. The target protein was analyzed using an AlphaImager 2200 gel image processing system (Protein Simple, CA, USA). The ratio of the 5’-NT band optical density to that of the β-actin band indicated the protein expression level.

### Statistical analysis

The SPSS 25.0 software (SPSS Inc., Chicago, IL, USA) was used for statistical analyses, and the data were expressed as mean ± standard error of the mean (SEM). One-way analysis of variance (ANOVA) was used to assess differences in 5'-NT expression and adenosine content between the groups. If the variance was homogeneous, a least significant difference test was used for pairwise comparison, and if the variance was not homogeneous, a Tamhane test was used for pairwise comparison. The MWT and TPT data of the rats were analyzed by means of repeated-measurement ANOVA, and P < 0.05 indicated a statistically significant difference.

## RESULTS

### Effect of EA on the expression of 5'-NT in CCI rats

**Figure 1.**
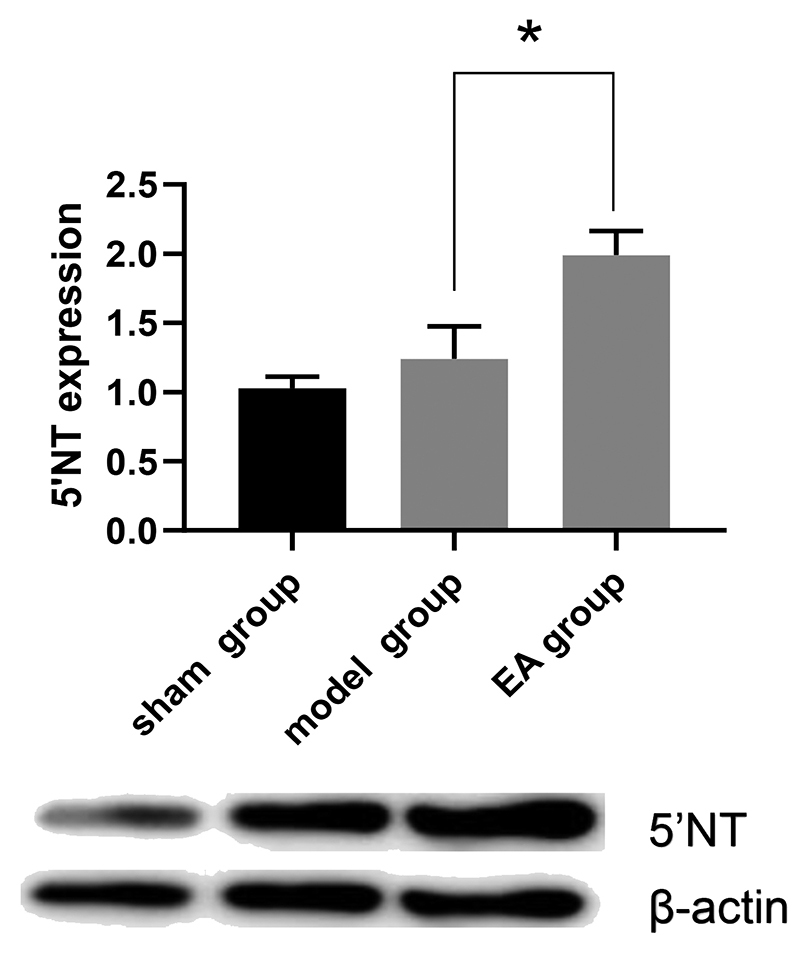
EA increased the expression of 5'-NT in CCI rats. (A): Representative western blots of 5'-NT in the L4-L6 spinal dorsal horn on day seven after CCI induction (B): Quantification analysis on the optical density of these bands. The values are presented as means ± SEM. EA group *vs.* model group (*P = 0.026).

CCI: chronic constriction injury; EA: electroacupuncture; SEM: standard error of the mean; 5-NT: ecto-5’-nucleotidase.

The 5’-NT content of the spinal cord of the rats in the EA group was significantly greater than that of the rats in the model group (P = 0.026) (Figure 1).

### AOPCP inhibited EA from upregulating adenosine content in the spinal cord of CCI rats

**Figure 2.**
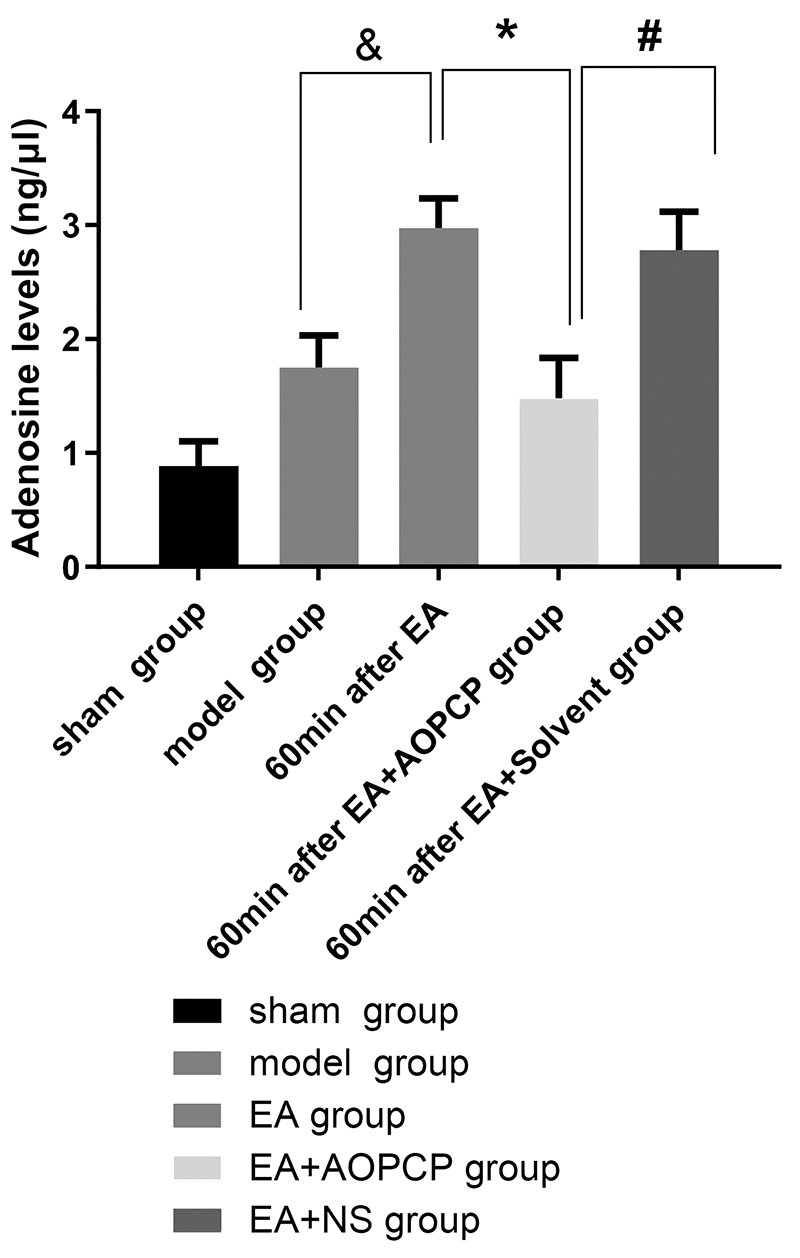
AOPCP can inhibit EA from upregulating the adenosine content in the spinal cord of CCI rats.The values are presented as means ± SEM. EA group vs. model group (^*^P = 0.018); EA + AOPCP group vs. EA group (^#^P = 0.002); EA + AOPCP group vs. EA + NS group (^&^P = 0.016).

AOPCP: α,β-methyleneadenosine 5'-diphosphate; CCI: chronic constriction injury; EA: electroacupuncture; SEM: standard error of the mean.

The adenosine content of the spinal cord of the rats in the EA group was significantly higher (P = 0.018) than that of the model group. Also. the adenosine content of the spinal cord of the rats in the model group was higher than that of the rats in the sham group. Compared with the EA and EA + NS groups, the EA + AOPCP group had significantly lower adenosine content in the spinal cord (P = 0.002 and P = 0.016, respectively). There was no significant difference in spinal adenosine content between the EA and EA + NS groups (P = 0.9630), thus indicating that the solvent of AOPCP (normal saline) did not affect the experimental results (Figure 2).

### AOPCP reversed the analgesic effect of EA on CCI rats

**Figure 3.**
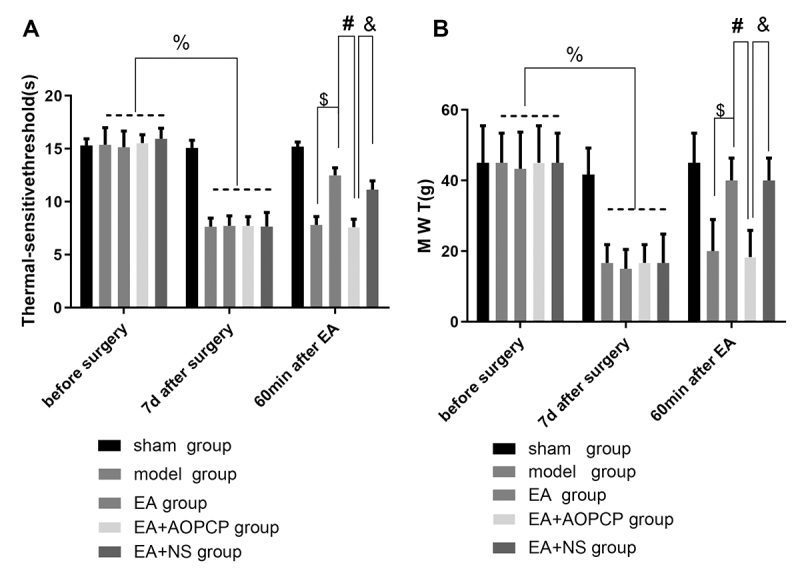
AOPCP can reverse the analgesic effect of EA in CCI rats. The MWT (A) and TPT (B) of the right hind paw at pre-surgery, 7 days post-surgery and 1 h post-EA are shown. The values are presented as means ± SEM. On the seventh day after modeling, the rats in each group, except for the sham group, were compared with those before modeling (^%^P < 0.0001 for A and B). At 60 min after EA, the following comparisons were conducted: EA group vs. model group (^$^P = 0.012 for A; ^$^P < 0.0001 for B); EA + AOPCP group vs. EA group (^#^P = 0.003 for A; ^#^P < 0.0001 for B); and EA + AOPCP group vs. EA + NS group (^&^P = 0.003 for A; ^&^P < 0.0001 for B).

AOPCP: α,β-methyleneadenosine 5'-diphosphate; CCI: chronic constriction injury; EA: electroacupuncture; MWT: mechanical withdrawal threshold; TPT: thermal pain threshold; SD: standard deviation.

On the seventh day after the model had been established, the MWT and TPT of the rats in all the groups, except the sham group, had decreased significantly, compared with those before modeling (P < 0.0001 for MWT and TPT), thus indicating that the models were successfully established in all groups. Compared with the MWT and TPT of the model group, those of the EA group were significantly higher at 60 min after EA (P = 0.012 for MWT; P < 0.0001 for TPT). In the EA group, compared with the MWT and TPT of the rats on the seventh day after modeling, they were significantly higher 60 min after EA (P = 0.003 for MWT; P < 0.0001 for TPT), thus indicating that EA had an analgesic effect on CCI rats. Compared with the EA and EA + NS groups, the EA + AOPCP group had significantly decreased MWT and TPT at 60 min after EA (P = 0.003 for MWT; P < 0.0001 for TPT). There was no significant difference in MWT or TPT between the EA and EA + NS groups (P = 0.9995 for MWT; P = 0.7489 for TPT), thus indicating that the solvent of AOPCP (normal saline) did not affect the experimental results (Figure 3).

### CCPA restored the analgesic effect of EA on CCI rats

**Figure 4.**
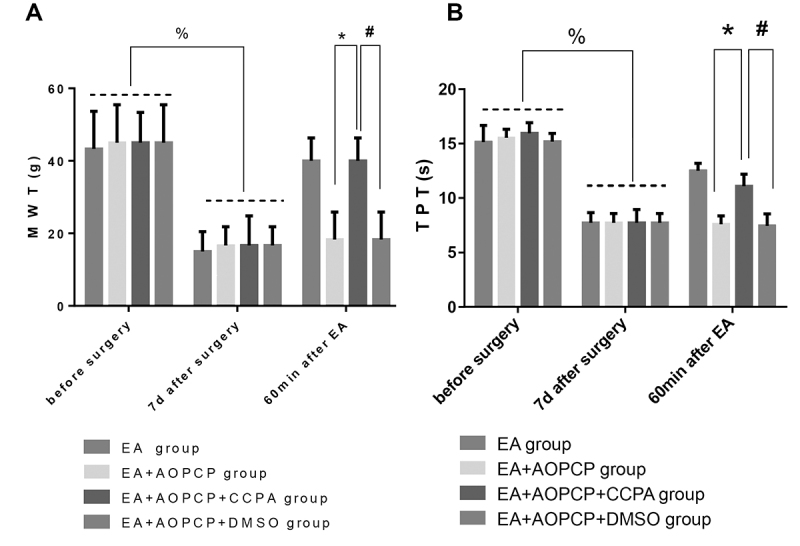
CCPA can restore the analgesic effect of EA in CCI rats. The MWT (A) and TPT (B) of the right hind paw at pre-surgery, 7 d post-surgery and 1 h post-EA are shown. The values are presented as means ± SEM. On the seventh day after modeling, the rats in each group, except for the sham group, were compared with those before modeling (^%^P < 0.0001 for A and B). At 60 min after EA, the following comparisons were conducted: EA + AOPCP group vs. EA + AOPCP + CCPA group (^*^P = 0.0003 for A; ^*^P < 0.0001 for B); and EA + AOPCP + DMSO group vs. EA + AOPCP + CCPA group (^#^P = 0.0003 for A; ^#^P < 0.0001 for B).

AOPCP: α,β-methyleneadenosine 5'-diphosphate; CCI: chronic constriction injury; CCPA: N6-cyclopentyladenosine; EA: electroacupuncture; MWT: mechanical withdrawal threshold; TPT: thermal pain threshold; SD: standard deviation.

On the seventh day after the model had been established, the MWT and TPT of the rats in each group had decreased significantly, compared with the values obtained before modeling (P < 0.0001 for both MWT and TPT), thus indicating that the models were established successfully in each group. Compared with the EA + AOPCP and EA + AOPCP + DMSO groups, the EA + AOPCP + CCPA group had significantly increased MWT and TPT levels at 60 min after EA (P = 0.0003 for MWT; P < 0.0001 for TPT). There was no statistical difference in MWT or TPT between the EA + AOPCP and EA + AOPCP + DMSO groups (P = 0.6867 for A; P = 0.7886 for B), which indicated that the solvent of CCPA (DMSO) did not affect the experimental results (Figure 4).

## DISCUSSION

At present, acupuncture is widely used for clinical treatment of pain. The purpose of central regulation by acupuncture is primarily to induce neurons to release neurotransmitters, neuromodulators or other chemical substances, such as endogenous opioids and adenosine, which have an impact on the body, into the brain tissue^9^^-^^11^. Among these, adenosine (which is a neurotransmitter) has received extensive attention in studies that focused on the mechanism of acupuncture analgesia. Adenosine mainly acts on adenosine receptors on the cell membrane, thus playing a biological function^12^. There are four adenosine receptor subtypes: A1, A2a, A2b and A3. Among these, adenosine A1 receptors are widely distributed in the spinal dorsal horn neurons, and activation of adenosine A1 receptors can inhibit the excitability of spinal neurons and achieve analgesic effects^13^. 

The most revered study on adenosine and acupuncture analgesia was published in *Nature Neuroscience* in 2010^14^. It claimed that the increase in adenosine content around the Zusanli acupoint in mice after EA was the main mechanism of acupuncture analgesia and that the analgesic effect of acupuncture was eliminated in adenosine A1 receptor knockout mice^14^. In addition, during human experiments, some researchers found that adenosine content could increase around the Zusanli acupoints, further corroborating the evidence that adenosine is involved in the mechanism for acupuncture analgesia^15^. It has also been shown that electrical stimulation increases the content of ATP and its metabolite, adenosine, in the thalamus^16^. Moreover, our own previous studies showed that adenosine content in the spinal cord of CCI rats can increase 60 min after EA, while silencing the adenosine A1 receptors can reverse the analgesic effect of EA^11^. However, how EA regulates adenosine production remains to be clarified. 

According to the existing research, 5’-NT is the most critical enzyme in the adenosine generation pathway^5^. In earlier studies, 5’-NT was found to be an enzyme with innate protective effects against lung injury^17^. Subsequent studies used 5’-NT knockout mice to establish a model for inflammatory pain and found that, compared with wildtype mice, knockout mice were more sensitive to pain and had significantly lower levels of adenosine^18^. 

In this experimental study, we first found that EA could upregulate 5'-NT expression in the spinal cord of CCI rats. Second, when AOPCP was used to inhibit the activity of 5'-NT, we found that the adenosine content in the spine in the EA + AOPCP group was significantly lower than that of the EA and EA + NS groups, thus indicating that AOPCP can reverse the upregulation effect of EA on adenosine content in the spinal cord of CCI rats. At the same time, the MWT and TPT levels in the EA + AOPCP group were significantly lower than those in the EA and EA + NS groups, which provided strong evidence that AOPCP could reverse the analgesic effect of EA on CCI rats. Lastly, we found that the MWT and TPT levels were significantly higher than those of rats treated with AOPCP alone, after administration of adenosine A1 agonist and AOPCP in CCI rats. Compared with the EA group, there was no significant difference in the MWT or TPT of rats in the EA + AOPCP + CCPA group, thus indicating that EA analgesia could still appeared again after supplementation with exogenous adenosine. Therefore, we hypothesized that EA may increase the level of adenosine and produce analgesic effects through upregulating the expression of 5'-NT in the spinal cord of CCI rats.

However, our study had some shortcomings: we only used AOPCP to inhibit the activity of 5'-NT; this could only demonstrate the involvement of 5'-NT in the analgesic effect of EA at the pharmacological level. If an adenovirus vector can be designed to knock down the expression of 5'-NT in rat spinal cords, we may be able to demonstrate the relationship between 5'-NT and EA analgesia at the gene level, which is considered more conclusive than pharmacological evidence.

In conclusion, we believe that EA can upregulate the expression of 5'-NT in the spinal cord of CCI rats and that AOPCP can significantly reverse the upregulation of adenosine content in the spinal cord of CCI rats and the analgesic effect of EA on CCI rats, while exogenous adenosine supplementation can restore the analgesic effect of EA. Therefore, we hypothesize that 5'-NT mediates the analgesic effect of EA in CCI rats.
